# Impact of Winter Holidays on Body Composition Measured via Bioelectrical Impedance Analysis: A Prospective Study

**DOI:** 10.3390/jcm14217566

**Published:** 2025-10-25

**Authors:** Ion-Vladut Udroiu, Alin Albai, Sandra Lazar, Adina Braha, Laura Gaita, Bogdan Timar, Alexandra Sima

**Affiliations:** 1 Doctoral School of Medicine, “Victor Babes” University of Medicine and Pharmacy, 300041 Timisoara, Romania; ion-vladut.udroiu@umft.ro (I.-V.U.); sandra.lazar@umft.ro (S.L.); 2 Department of Diabetes, “Pius Brinzeu” Emergency Hospital, 300723 Timisoara, Romania; braha.adina@umft.ro (A.B.); gaita.laura@umft.ro (L.G.); bogdan.timar@umft.ro (B.T.); sima.alexandra@umft.ro (A.S.); 3 Centre for Molecular Research in Nephrology and Vascular Disease, “Victor Babes” University of Medicine and Pharmacy, 300041 Timisoara, Romania; 4 Second Department of Internal Medicine, “Victor Babes” University of Medicine and Pharmacy, 300041 Timisoara, Romania; 5 First Department of Internal Medicine, “Victor Babes” University of Medicine and Pharmacy, 300041 Timisoara, Romania; 6 Department of Hematology, Emergency Municipal Hospital, 300254 Timisoara, Romania

**Keywords:** winter holidays, body composition, bioimpedance analysis, fat mass, visceral fat area

## Abstract

**Background/Objectives:** The winter holiday period is often associated with lifestyle changes that can affect body composition. This study aimed to evaluate short-term changes in body composition and anthropometric indices over the winter holidays. **Methods:** A total of 168 adults (126 women and 42 men) were assessed before (December) and after (January) the holidays using bioelectrical impedance analysis (InBody 770) and standard anthropometric measurements. Participants also completed a Healthy Eating Assessment questionnaire to evaluate their dietary habits during this period. **Results:** After the holiday, statistically significant increases were observed in weight (68.55 → 69.70 kg), body fat mass (20.60 → 21.15 kg), visceral fat area (95.40 → 97.60 cm^2^), and waist circumference (84.30 → 85.08 cm). Men showed greater gains in weight and fat-related parameters compared to women. Participants who reported healthier dietary behaviors had smaller increases in fat mass and anthropometric measures. **Conclusions:** These findings suggest that even brief holiday periods can lead to measurable gains in weight and body fat and, if repeated over time, may contribute to the development of obesity.

## 1. Introduction

Obesity is a chronic disease characterized by an excess of adipose tissue that may damage health. Although the scientific literature on obesity is expanding quickly, the prevalence of obesity is increasing even faster. According to the World Health Organization (WHO), in 2022, approximately 2.5 billion adults were overweight (43%), and 890 million adults were obese (16%) [1]. Without significant interventions, by 2030, these percentages will increase rapidly, with an estimated 50% of adults being overweight and 20% being obese [2].

Obesity shortens life expectancy and increases the risk of several non-communicable diseases [2,3,4,5]. But not all individuals with obesity face the same risk. Some very obese patients may still have a neutral risk profile, while overweight individuals may already have conditions like type 2 diabetes or heart disease [6]. One possible explanation for these differences in risk is the distribution of body fat. Even if subcutaneous adipose tissue (SAT) plays an important role, visceral adipose tissue (VAT) is especially problematic because of its association with diabetogenic, atherogenic, prothrombotic, and proinflammatory metabolic states [6,7].

Identifying the exact causes of obesity remains a significant challenge. There are a lot of risk factors involved, many of which are interconnected. Even if the genetic factor is non-modifiable and contributes to an individual’s susceptibility to obesity, obesity is primarily caused by an imbalance between energy intake and energy expenditure [8]. The key contributors are the consumption of energy-dense foods and a lack of physical activity. However, this imbalance can also be influenced by other factors like environment, socioeconomic status, certain drugs, medical conditions, and stress [9]. Moreover, there is an important link between body mass index (BMI), age, and sex in determining differences in body composition. Evidence shows that with age, there are gradual increases in body fat mass (BFM) and decreases in skeletal muscle mass (SMM) [10]. In terms of sex differences, men typically have higher fat-free mass (FFM) and lower BFM than women [11]. Additionally, major lifestyle disruptions, such as those caused by the COVID-19 pandemic, have been associated with reductions in FFM and SMM, particularly in young men [12].

Considering all of this, many risk factors may be amplified during some periods of the year. An average adult gains around 0.5–1 kg of weight per year, but is this gain linear or do they gain more in certain periods of the year [13]?

Different countries celebrate various holidays at the end of the year, but they all share a common characteristic: increased consumption of specific foods [14]. Also, social gatherings and cultural traditions create an environment where weight gain becomes more likely. Several factors during this period can encourage overconsumption, such as the availability of food, longer mealtimes, larger portions than usual, and eating with other people [15]. De Castro (1995) observed that people tend to eat 44% more when other people are present [16]. Also, the amount of food consumed is positively correlated with the number of people present. Levitsky (2002) found that larger portion sizes lead people to eat more [17]. These findings were also supported by Rolls et al. (2002) [18].

Given these considerations, this study aimed to evaluate how the winter holiday period impacts weight and body composition, with a particular focus on visceral fat area (VFA) as estimated via bioelectrical impedance analysis (BIA).

## 2. Materials and Methods

This was an observational, longitudinal study conducted at the Diabetes Center of the Emergency County Hospital “Pius Brînzeu” in Timișoara, Romania.

Participants were recruited voluntarily through a post on social media, and those interested contacted us. The inclusion criteria were over 18 years and availability to come to both measurement visits. Participants were excluded if they were pregnant or had implanted pacemakers.

A total of 168 participants were recruited and measured at two time points: the beginning of December 2024 and January 2025, right after the holidays. In Romania, December represents an extended festive period that typically includes several public holidays and celebrations (National Day on 1st December, Saint Nicholas on December 6th, Christmas, Saint Stephen on 27th December, New Year, Epiphany on 6th January, and Saint John the Baptist on 7th January). Most people take a vacation during this time, resulting in a consistent holiday period across the country. This time frame was the same for all participants, and both visits were scheduled before and immediately after this period.

All participants gave their written informed consent before taking part in the study. The research protocol was approved by the Ethics Committee of the Emergency County Hospital “Pius Brînzeu” in Timișoara, Romania (approval no. 508/25 November 2024). The study respected the ethical guidelines of the Declaration of Helsinki (2013), and all personal information was kept confidential, following General Data Protection Regulation (GDPR) standards.

Height and body weight were measured using a digital scale with an integrated stadiometer (Pegaso electronic body scale, GIMA S.p.A., Gessate, Italy). Body composition was assessed using BIA with the InBody 770 device (InBody Co., Ltd., Seoul, Republic of Korea). Measurements were performed in standardized conditions: in the morning, in a fasted state, with light clothing and with no recent physical activity. Waist circumference (WC) and hip circumference (HC) were also measured using a flexible measuring tape. The measurements followed WHO recommendations: waist circumference was taken at the approximate midpoint between the top of the iliac crest and the lower margin of the last palpable rib, while hip circumference was measured around the widest portion of the buttocks [19].

To evaluate the quality of dietary habits during the winter holiday period, participants completed the Healthy Eating Assessment questionnaire, a standardized tool published by the Government of Northwest Territories (Canada) in January 2017 [20]. This assessment consists of 10 questions, and it covers various aspects of eating behaviors. It evaluates the perception of participants for their overall healthy eating habits and the frequency of consuming specific foods (e.g., fried foods, fast food, fruits, vegetables, sugary drinks, meat, dairy products, etc.) during the winter holiday. Each item is scored on a scale from 1 (poor) to 5 (excellent), with a total score ranging from 10 to 50 points. The total score is interpreted as follows: 10–19 points indicate needs improvement, 20–29 fair, 30–39 good, and 40–50 excellent eating behavior. The questionnaire was translated and adapted for use with Romanian participants and was completed by all participants at the second visit, after the winter holiday period.

Statistical analysis was performed with MedCalc^®^ Statistical Software version 23.1.3 (MedCalc Software Ltd., Ostend, Belgium; https://www.medcalc.org (accessed on 15 March 2025)). The Shapiro-Wilk test was used to evaluate the normal distribution of continuous numerical variables. Normally distributed variables are presented as the mean ± standard deviation (SD), while non-normally distributed variables are shown as the median and interquartile range (IQR). Paired *t*-tests or Wilcoxon tests were used to evaluate participant differences between visits (T1 vs. T2), depending on variable distribution. Unpaired *t*-tests or Mann–Whitney U tests were applied to assess group differences (men vs. women, rural vs. urban). Spearman’s correlation coefficients were calculated to examine the associations between changes in body fat mass (ΔBFM) and changes in body weight (ΔWeight), between ΔVFA and ΔWeight, and between ΔVFA and ΔBFM. Following that, simple linear regression analyses were performed to evaluate the predictive relationships between these variables. Statistical significance was considered at *p* ≤ 0.05. All tests were conducted with a 95% confidence level. Given the exploratory and observational nature of this study, no formal power analysis was performed. For the same reason, no formal correction for multiple comparisons was applied. The potential risk of type I error was considered when interpreting the results. Effect sizes were not systematically calculated for all paired comparisons because the main aim of the study was to describe within-subject changes rather than to test predefined hypotheses. However, the strength of associations between parameters was expressed through Spearman’s correlation coefficients (ρ) and coefficients of determination (R^2^) obtained from regression analyses.

## 3. Results

Baseline characteristics of the participants are presented in Table 1. The study included 168 individuals with a median age of 30 years (IQR 27–44). The mean height was 168.01 ± 8.82 cm.

Most of our participants were women (126/168, 75%). No significant difference was observed in age between women (median 30 years) and men (median 28.5 years). The majority of men were overweight, with a BMI of 25.8 kg/m^2^ (IQR 24.3–27.7), compared to women (BMI 24.0 kg/m^2^ [IQR 21.3–27.8], *p* = 0.015), as presented in Table 2. Men also showed significantly greater body cell mass (BCM), FFM, SMM, total body water (TBW), intracellular water (ICW), and extracellular water (ECW) than women (all *p* < 0.001). 

Conversely, women showed significantly higher BFM (21.85 kg [IQR 16.2–28.4] vs. 16.95 kg [IQR 14.6–22.3]; *p* = 0.004) and percentage of body fat (PBF) (33.95% ± 7.68 vs. 22.24% ± 6.63; *p* < 0.001). VFA and visceral fat level (VFL) were also significantly higher in women (*p* = 0.002 for both). Although HC did not differ significantly between groups (*p* = 0.059), WC, waist-to-hip ratio (WHR), and waist-to-height ratio (WHtR) were significantly higher in men than in women (all *p* < 0.05). The baseline characteristics of participants by sex are presented in Table 2.

**Figure 1:**
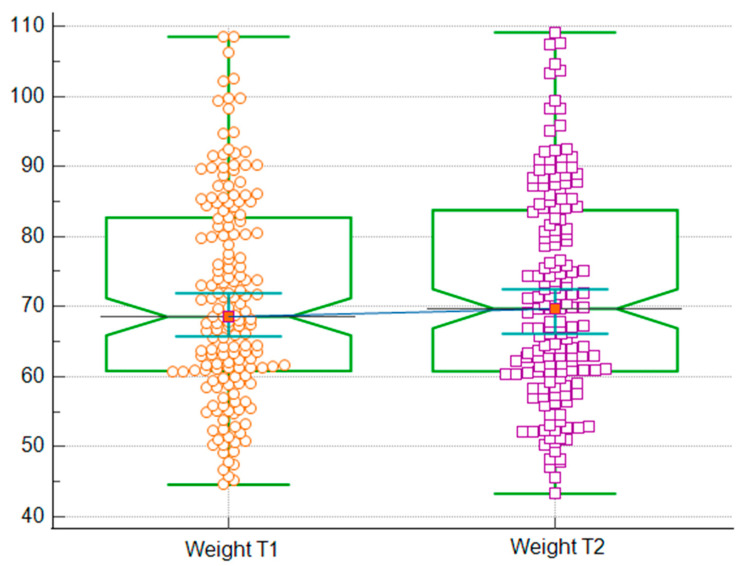
Comparison of body weight before (T1) and after (T2) the winter holiday period. Orange circles represent individual body weight values before the winter holiday (T1), and purple squares represent values after the winter holiday (T2).

**Figure 2:**
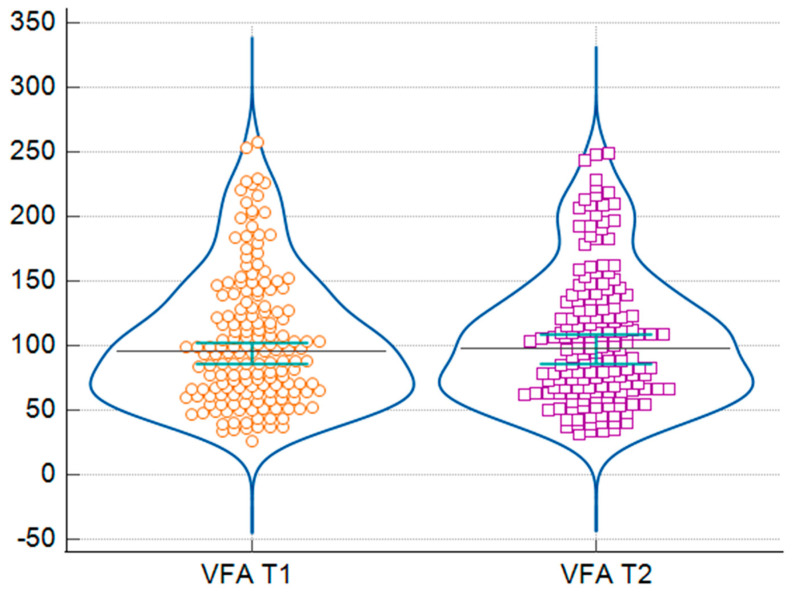
Distribution of visceral fat area (VFA) before (T1) and after (T2) the winter holiday period. Orange circles represent individual VFA values before the winter holiday (T1), and purple squares represent values after the winter holiday (T2).

As seen in Table 3, after the winter holidays, most participants showed statistically significant changes in weight, BMI, BFM, PBF, VFA, VFL, WC, HC, WHR, and WHtR (all *p* < 0.05). Specifically, weight increased from 68.55 kg to 69.70 kg (*p* = 0.003) (Figure 1), and BMI increased from 24.55 to 24.70 kg/m^2^ (*p* = 0.004). BFM increased from 20.60 kg to 21.15 kg (*p* < 0.001), which also led to a significant increase in PBF, from 30.65% to 30.99% (*p* = 0.0006). Both VFA and VFL were higher after the holidays, with VFA increasing from 95.40 cm^2^ to 97.60 cm^2^ (*p* < 0.001) (Figure 2) and VFL also showing a significant increase (*p* < 0.001). Both anthropometric indices measured in our study, WC and HC, showed statistically significant changes. WC increased from 84.30 to 85.08 cm (*p* < 0.001) and, even if the median stayed the same for HC, the *p*-value was 0.0101. These changes also led to significant changes in the WHR, from 0.82 to 0.83 (*p* < 0.001), and in WHtR, from 0.502 to 0.507 (*p* < 0.001). No significant changes were observed in BCM, FFM, SMM, TBW, ECW, ICW, and phase angle (all *p* > 0.05).

**Figure 3:**
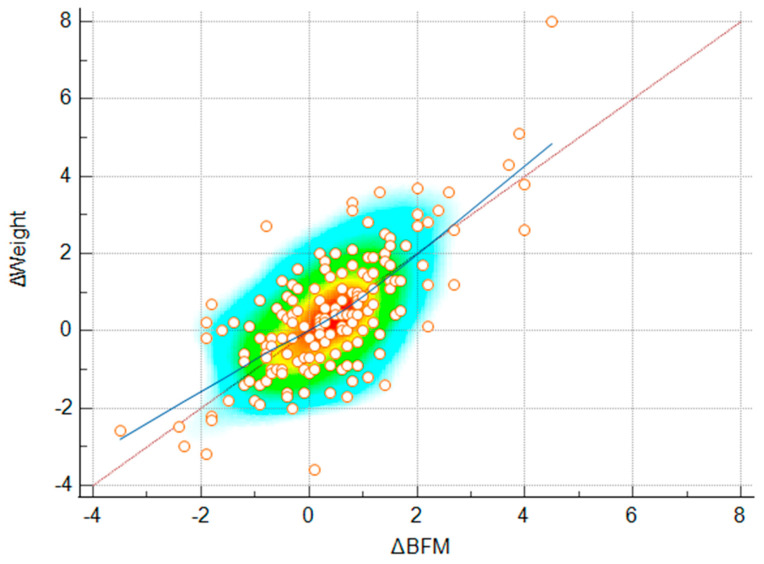
Spearman rank correlation between changes in body fat mass (ΔBFM) and changes in body weight (ΔWeight) during the winter holiday period. The background color gradient represents the density of data points, with warmer colors (yellow–orange) indicating higher concentrations. Circles represent individual data points. The blue line illustrates the linear trend fitted across all data points, while the red diagonal represents the line of identity.

**Figure 4:**
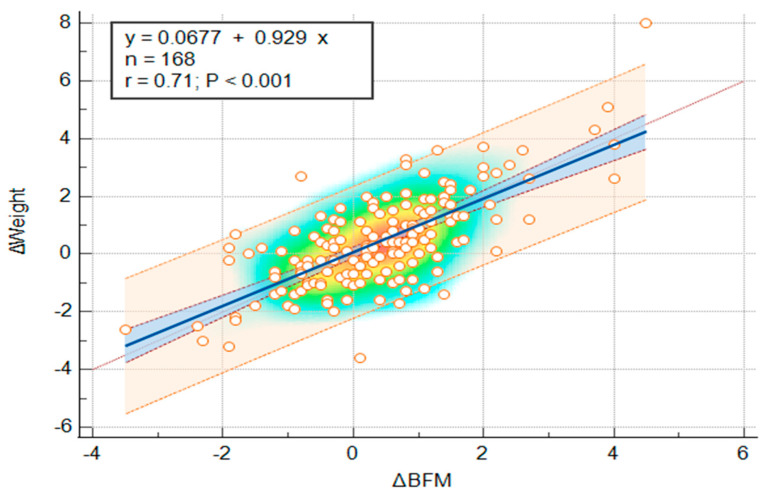
Simple linear regression illustrating the relationship between changes in body fat mass (ΔBFM) and changes in body weight (ΔWeight) during the winter holiday period. The background color gradient represents the density of data points, with warmer colors (yellow–orange) indicating higher concentrations. Circles represent individual data points. The blue line indicates the linear regression, and the shaded area represent the 95% confidence interval.

A Spearman rank correlation analysis was performed to evaluate the association between ΔBFM and ΔWeight during the winter holiday period. The analysis presented a strong, positive, and statistically significant correlation between ΔBFM and ΔWeight (ρ = 0.644, *p* < 0.001) (Figure 3). Following this, a simple linear regression analysis was performed to evaluate the predictive relationship between ΔBFM and ΔWeight. The regression model was statistically significant (R^2^ = 0.508, *p* < 0.001), indicating that approximately 50.8% of the variance in weight change was explained by changes in BFM. This suggests that for each 1 kg increase in body weight, BFM increased by approximately 0.93 kg on average (Figure 4).

**Figure 5:**
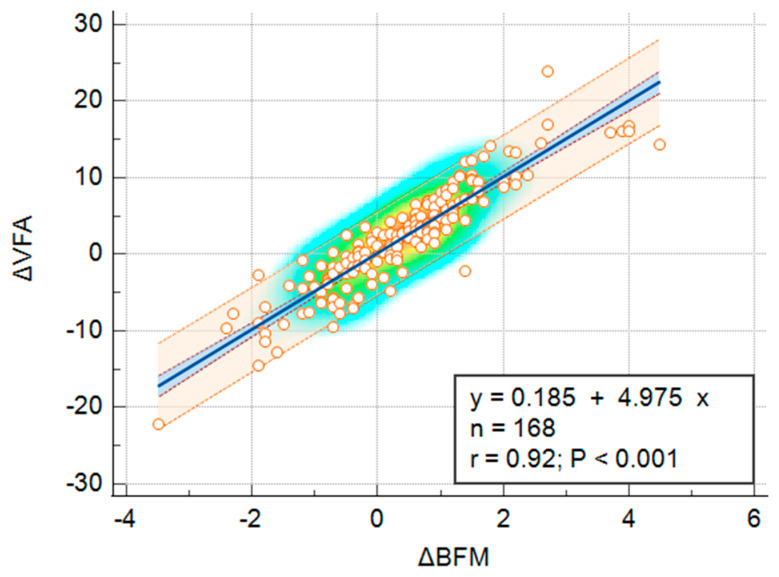
Simple linear regression illustrating the relationship between changes in visceral fat area (ΔVFA) and changes in body fat mass (ΔBFM) during the winter holiday period. The background color gradient represents the density of data points, with warmer colors (yellow–orange) indicating higher concentrations. Circles represent individual data points. The blue line indicates the linear regression, and the shaded area represent the 95% confidence interval.

Spearman rank correlation analyses demonstrated a strong, positive, and statistically significant association between Δ VFA and Δ Weight (ρ = 0.607, *p* < 0.001), as well as an even stronger correlation between Δ VFA and Δ BFM (ρ = 0.928, *p* < 0.001). Linear regression analyses showed that Δ Weight significantly predicted Δ VFA (y = 0.984 + 2.625x, *r* = 0.63, R^2^ = 0.3964, *p* < 0.001), explaining approximately 39.6% of the variance. An even stronger predictive relationship was found between Δ BFM and Δ VFA (y = 0.185 + 4.975x, *r* = 0.92, R^2^ = 0.8381, *p* < 0.001), accounting for 83.8% of the variance (Figure 5).

Table 4 presents a comparison of changes in anthropometric and body composition parameters between sexes during the winter holiday. Significant sex differences were observed in the changes for weight, BMI, BFM, PBF, VFL, and WC (all *p* < 0.05), with men showing higher increases than women. Although the absolute changes in VFL and WC were small, they reached statistical significance. VFA tended to increase more in men during the holiday, but this difference did not reach statistical significance (*p* = 0.064). No parameter showed greater increases in women, and no significant sex differences were observed in BCM, FFM, SMM, HC, phase angle, WHR, WHtR, or any of the body water compartments (all *p* ≥ 0.05).

No statistically significant differences were found in the changes of anthropometric or body composition parameters between participants who spent the holidays in rural (*n* = 51, 30.4%) versus urban (*n* = 117, 69.6%) environments (all *p* ≥ 0.05). The median weight change was 0.2 kg (IQR −0.6–1.35) in the rural environment group and 0.3 kg (IQR −0.7–1.3) in the urban environment group.

**Figure 6:**
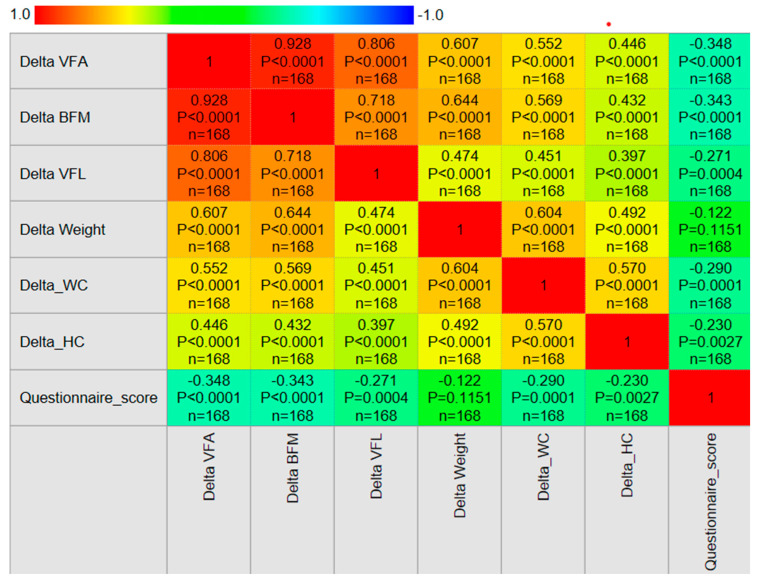
Spearman correlogram showing strength and direction of correlations between changes in anthropometric and body composition parameters, including questionnaire score (*n* = 168). Colors indicate magnitude and direction of correlations; Abbreviations: VFA, visceral fat area; BFM, body fat mass; VFL, visceral fat level; WC, waist circumference; HC, hip circumference. Note: “Delta” indicates the change between pre- and post-holiday measurements.

The median questionnaire score was 32.0 points (IQR 29.0–35.0), suggesting overall good dietary behaviors during the holiday period. Spearman correlation analysis revealed that higher questionnaire scores were negatively correlated with ΔVFA (ρ = −0.348, *p* < 0.001), ΔBFM (ρ = −0.343, *p* < 0.001), Δ VFL (ρ = −0.271, *p* < 0.001), ΔWC (ρ = −0.290, *p* < 0.001), and ΔHC (ρ = −0.230, *p* = 0.003) (Figure 6). These results indicate that participants who reported healthier behaviors had smaller increases in fat-related parameters.

## 4. Discussion

This Romanian population-based study evaluated the short-term impact of the winter holiday period on body composition and anthropometric parameters.

Obesity is a significant public health concern affecting a growing number of people around the world, which has intensified research into its causes. One factor that has received particular attention is the role of holidays.

Holidays seem to promote weight gain in adults, according to a narrative review titled Effect of the Holiday Season on Weight Gain, which included 15 publications. In these studies, significant weight increases were consistently observed during the holiday period, ranging from 0.4 to 0.9 kg (*p* < 0.05) [21]. More specifically, a study conducted in Maryland, United States, concluded that their participants gained on average 0.37 kg during the holiday period [22]. Another international study, which evaluated the impact of the Christmas period in three countries, the United States, Japan, and Germany, found weight increases of 0.4% in the United States (*p* < 0.001), 0.6% in Germany (*p* < 0.001), and 0.5% in Japan (*p* = 0.005) [14]. Another study conducted in the United Kingdom, which evaluated changes in nutritional status in adults over Christmas, concluded that a mean weight gain of 0.93 kg occurred [23]. More recently, Abdulan et al. (2025) [24] conducted a systematic review including ten studies with a total of 4627 participants and found that most individuals gained weight during the holiday season. Most of the weight gained during these periods of the year was maintained at follow-up [24]. Viñuela et al. (2023) showed that even university students had body weight increase from 59.6 to 60.2 kg (*p* = 0.010) over the Christmas holiday period [25]. Bhutani et al. (2020) found that although the mean body weight change during the holidays was modest (0.41 kg), increased food intake was the most likely cause of this gain [26].

While these studies consistently demonstrate modest but significant increases in body weight during the winter holiday period, studies from other countries are required, considering that the vast majority of the existing research was conducted in the United States and the United Kingdom [21].

Our results are in line with previous studies, confirming that the winter holiday period is associated with statistically significant increases in body weight. However, our study provides further information by analyzing changes in BFM, VFA, and other body composition parameters in addition to weight, which were not routinely evaluated in earlier research. The main findings in our study revealed statistically significant increases in body weight (from 68.55 kg to 69.70 kg, *p* = 0.003), BMI (from 24.55 to 24.70 kg/m^2^, *p* = 0.004), BFM (from 20.60 kg to 21.15 kg, *p* < 0.001), PBF (from 30.65% to 30.99%, *p* < 0.001) during the holidays. VFA increased from 95.40 cm^2^ to 97.60 cm^2^ (*p* < 0.001), suggesting that even a short holiday period can lead to meaningful changes in VFA. Additionally, statistically significant increases were observed in WC, HC, WHR, and WHtR. Conversely, no significant changes were observed in FFM, SMM, phase angle, or body water compartments.

These results underscore the fact that the winter holiday period is not limited to only body weight increase but comes with important changes in body fat. This statement is supported by our Spearman rank correlation analysis, which showed a strong and statistically significant correlation between ΔBFM and ΔWeight, suggesting that for each 1 kg increase in body weight, body fat mass increased by approximately 0.93 kg on average. However, there were only slight changes in parameters like body water compartments and FFM, indicating that almost all the weight gain was due to increases in fat tissue. Although the observed changes were statistically significant, the absolute increases were modest. Still, when such small gains recur during successive holiday periods, they may gradually accumulate and lead to higher levels of body fat.

Regardless of changes in total fat or SAT, VAT is strongly associated with a variety of cardiometabolic abnormalities [27]. Excess VAT is associated with insulin resistance, hyperinsulinemia, glucose intolerance, type 2 diabetes mellitus, dyslipidemia characterized by high triglycerides and small dense LDL particles, systemic inflammation, endothelial dysfunction, and an increased risk of thrombosis [28,29,30,31,32].

In our study, participants had an average increase of 2.2 cm^2^ in VFA during the short winter holiday period. ΔVFA showed a very strong positive correlation with ΔBFM (ρ = 0.928, *p* < 0.001) and a strong correlation with ΔWeight (ρ = 0.607, *p* < 0.001), suggesting that increases in VFA are linked to weight gain and overall fat mass gain during the winter holiday period. Linear regression analysis showed that changes in BFM explained most of the variation in VFA (R^2^ = 0.8381), suggesting that gaining fat during the holidays is closely tied to increases in VFA. This pattern was also seen in the VFL parameter. Although the median VFL level remained the same (level 9) before and after the holiday period, the Wilcoxon test showed a very significant *p*-value (*p* < 0.001). This indicates that, despite an unchanged central tendency, the distribution of individual changes shifted significantly, confirming that VFL increased in many participants. These results emphasize that even small increases in VFA have clinical significance, considering the established link between VAT and cardiometabolic disorders [6,7,27,33].

WC and WHR are some of the most used proxies for estimating VAT, and both correlate strongly with VAT levels [34,35]. Abdominal obesity, as assessed by these parameters, is correlated with the risk of CVD events [27,36]. According to previous research, each 1 cm increase in WC is associated with a 2% increase in the risk of CVD, while a 0.01 increase in WHR leads to a 5% increase in risk [36]. In our study, WC increased significantly from 84.30 to 85.08 cm (*p* < 0.001), and WHR also increased from 0.82 to 0.83 (*p* < 0.001), emphasizing how quickly even small lifestyle changes can have an impact on cardiometabolic risk markers. HC showed a stable median (102.00 cm before and after the holiday period), but the change was statistically significant (*p* = 0.010), indicating subtle and consistent shifts in individual participants. WHtR increased from 0.502 to 0.507 (*p* < 0.001), and considering the fact that WHtR is a widely recognized index of central obesity and a strong predictor of cardiometabolic risk, this finding supports the idea that the winter holiday period can result in measurable and clinically relevant changes in central fat distribution [37,38].

Significant sex differences were observed during the winter holidays. Men showed greater increases in weight and fat-related parameters than women. They gained nearly four times more weight than women (0.75 kg vs. 0.20 kg). These differences were consistent across multiple fat-related parameters, while no significant differences were seen in lean mass compartments (FFM, SMM, TBW, ICW, and ECW). This indicates that the gained weight in men was predominantly fat. This pattern may reflect general behavioral trends previously described in the literature. Alcohol consumption increases during the holidays, and men tend to consume more alcohol than women [39,40]. Also, women tend to be more careful with their dietary choices [41]. However, since the questionnaire did not include questions regarding alcohol consumption, its role in explaining the observed differences remains speculative. No significant differences were found between participants from rural and urban environments, suggesting that the changes in body composition during the holiday period occur similarly regardless of where people live.

Our results also show the effect of adopting healthy eating behaviors during the winter holiday. Participants who reported healthier dietary habits, as assessed by the Healthy Eating Assessment questionnaire, showed smaller increases in BFM, VFA, VFL, WC, and HC. Those with lower questionnaire scores, meaning worse dietary behavior, had greater gains in these parameters. This supports the validity of this questionnaire and highlights its usefulness as a research tool. Also, these results reinforce the idea that dietary habits influence the direction of body composition changes during the winter holiday.

Several limitations of this study should be noted. First, participants were recruited voluntarily through a post on social media, which may have introduced selection bias. This may have favored individuals more interested in body composition and health behaviors. Also, because more women contacted us (75%) for the recruitment, there was an imbalance between sexes, which limits the generalizability of the results. Second, participants were aware of the primary aim of the study, and they knew that the second measurement would be after the winter holidays. This may have changed their behavior. Third, most participants were from western Romania, and considering regional differences in holiday traditions and foods, results may not be generalizable. Fourth, the Healthy Eating Assessment questionnaire relies on self-reported data. Additionally, it is not validated in the Romanian population and does not include questions regarding alcohol consumption, which is probably an important factor during the holiday period. Physical activity and sleep are important lifestyle factors and weren’t evaluated. They could act as confounding variables influencing body composition changes in the studied period. Finally, body composition was measured using the validated bioelectrical impedance device InBody 770, but like other BIA methods, it has its limitations in precision compared to gold-standard techniques such as DXA [42]. Recent studies have shown that multi-frequency BIA systems, including the InBody 770, demonstrate high reliability and good agreement with DXA in healthy adults. Some reports indicate that BIA may overestimate FFM in athletes, while others show good concordance for total and regional body composition compared with DXA [42,43,44,45]. However, BIA has lower precision in estimating visceral fat compared with imaging methods such as DXA, MRI, or CT [44,46,47]. Overall, these findings support the suitability of BIA for assessing short-term changes in body composition. Under real-life conditions, the use of DXA, MRI, or CT would be impractical or ethically questionable for repeated measurements for the purposes of the current study. Future research should include a more balanced and randomly selected sample, and also evaluate key lifestyle factors like physical activity, sleep, and alcohol use.

## 5. Conclusions

This study showed that body weight, fat mass, and waist circumference increased statistically significantly during the winter holiday period, with greater changes observed in men compared to women. Although these changes were modest, they occurred over a short period, emphasizing the vulnerability of this time of year. When accumulated with other similar periods, such changes may contribute to gradual weight gain over time and, if sustained, could play a role in obesity development. Visceral fat area also increased statistically significantly, suggesting that repeated similar periods may, in the long term, contribute to a higher cardiometabolic risk. Future research should include longitudinal follow-up to investigate if the weight and visceral fat area gained during the winter holiday are maintained and compare these changes with other vulnerable periods across the year. These findings support the implementation of preventive strategies for the winter holiday period to minimize short-term gains and to encourage healthier lifestyle behaviors.

## Figures and Tables

**Table 1:** Baseline characteristics of the study participants (*n* = 168).

Variable	Value
Age (years)	30 (27–44)
Height (cm)	168.01 ± 8.82
Weight (kg)	68.55 (60.8–82.7)
BMI (kg/m^2^)	24.55 (21.6–27.8)
BCM (kg)	30 (26.6–35.25)
BFM (kg)	20.6 (15.35–26.8)
PBF (%)	30.65 ± 8.87
FFM (kg)	46.15 (40.95–54.15)
SMM (kg)	25.3 (22.2–30.1)
TBW (l)	33.9 (30–39.7)
ECW (l)	12.8 (11.45–15.05)
ICW (l)	20.95 (18.6–24.6)
VFA (cm^2^)	95.4 (64.35–132.5)
VFL (level)	9 (6–13)
Phase Angle (°)	5.10 (4.8–5.9)
WC (cm)	84.30 (±13.10)
HC (cm)	102 (95.5–107.5)
WHR	0.82 (±0.082)
WHtR	0.502 (±0.075)

Abbreviations: BMI, body mass index; BCM, body cell mass; BFM, body fat mass; PBF, percent body fat; FFM, fat-free mass; SMM, skeletal muscle mass; TBW, total body water; ECW, extracellular water; ICW, intracellular water; VFA, visceral fat area; VFL, visceral fat level; WC, waist circumference; HC, hip circumference; WHR, waist-to-hip ratio; WHtR, waist-to-height ratio. Values are expressed as the mean ± SD for normally distributed variables and as the median (IQR) for non-normally distributed variables.

**Table 2:** Baseline characteristics of participants by sex.

Variable	Women (*n* = 126)	Men (*n* = 42)	*p*-Value
Age (years) ^b^	30.0 (27.0–46.0)	28.5 (26.0–33.0)	0.088 ns
Height (cm) ^a^	164.0 ± 6.928	178.5 ± 5.410	<0.001 ***
Weight (kg) ^b^	63.85 (57.6–75.1)	84.35 (73.6–89.5)	<0.001 ***
BMI (kg/m^2^) ^b^	24.0 (21.3–27.8)	25.8 (24.3-27.7)	0.015 *
BCM (kg) ^a^	28.444 ± 3.6284	41.94 ± 5.2218	<0.001 ***
BFM (kg) ^b^	21.85 (16.2–28.4)	16.95 (14.6–22.3)	0.004 **
PBF (%) ^a^	33.95 ± 7.683	22.240 ± 6.633	<0.001 ***
FFM (kg) ^a^	43.920 ± 5.6816	63.702 ± 7.8308	<0.001 ***
SMM (kg) ^a^	23.896 ± 3.2999	36.183 ± 4.7400	<0.001 ***
TBW (l) ^a^	32.186 ± 4.1671	46.693 ± 5.7472	<0.001 ***
ECW (l) ^b^	12.25 (11.2–13.3)	17.5 (15.9–18.5)	<0.001 ***
ICW (l) ^a^	19.858 ± 2.5304	29.286 ± 3.6456	<0.001 ***
VFA (cm^2^) ^b^	100.55 (66.9–143.2)	76.25 (59.2–102.9)	0.002 **
VFL (level) ^b^	10.0 (6.0–14.0)	7.0 (5.0–10.0)	0.002 **
Phase Angle (°) ^b^	5.0 (4.7–5.3)	6.4 (5.9–6.7)	<0.001 ***
WC (cm) ^a^	81.62 (± 13.01)	92.32 (± 9.74)	<0.001 ***
HC (cm) ^b^	100.0 (94–108)	103.0 (100–107)	0.059 ns
WHR ^a^	0.803 (± 0.076)	0.890 (± 0.060)	<0.001 ***
WHtR ^b^	0.480 (0.440–0.538)	0.522 (0.478–0.547)	0.017 *

^a^ *T*-test; ^b^ Mann–Whitney test; Abbreviations: BMI, body mass index; BCM, body cell mass; BFM, body fat mass; PBF, percent body fat; FFM, fat-free mass; SMM, skeletal muscle mass; TBW, total body water; ECW, extracellular water; ICW, intracellular water; VFA, visceral fat area; VFL, visceral fat level; WC, waist circumference; HC, hip circumference; WHR, waist-to-hip ratio; WHtR, waist-to-height ratio; ns = not significant *p* ≥ 0.05, * = statistically significant *p* < 0.05, ** = highly statistically significant *p* < 0.01, *** = very highly statistically significant *p* < 0.001. Values are expressed as the mean ± SD for normally distributed variables and as the median (IQR) for non-normally distributed variables.

**Table 3:** Changes in body composition and anthropometric parameters before (T1) and after (T2) the winter holidays.

Variable	Before (T1)	After (T2)	*p*-Value
Weight (kg) ^b^	68.55 (60.80–82.70)	69.70 (60.75–83.75)	0.003 **
BMI (kg/m^2^) ^b^	24.55 (21.60–27.80)	24.70 (21.95–28.10)	0.004 **
BCM (kg) ^b^	30.0 (26.6–35.25)	30.1 (26.6–35.65)	0.804 ns
BFM (kg) ^b^	20.60 (15.35–26.80)	21.15 (15.65–26.70)	<0.001 ***
PBF (%) ^a^	30.65 (±8.87)	30.99 (± 8.86)	<0.001 ***
FFM (kg) ^b^	46.15 (40.95–54.15)	46.30 (40.95–54.60)	0.736 ns
SMM (kg) ^b^	25.30 (22.20–30.10)	25.45 (22.20–30.50)	0.611 ns
TBW (l) ^b^	33.90 (30.00–39.70)	33.80 (30.0–40.00)	0.682 ns
ECW (l) ^b^	12.80 (11.45–15.05)	12.90 (11.40–15.20)	0.596 ns
ICW (l) ^b^	20.95 (18.60–24.60)	21.05 (18.60–24.90)	0.679 ns
VFA (cm^2^) ^b^	95.40 (64.35–132.50)	97.60 (66.75–135.45)	<0.001 ***
VFL (level) ^b^	9.00 (6.00–13.00)	9.00 (6.00–13.00)	<0.001 ***
Phase Angle (°) ^b^	5.10 (4.80–5.90)	5.20 (4.80–5.90)	0.689 ns
WC (cm) ^a^	84.30 (±13.10)	85.08 (± 13.20)	<0.001 ***
HC (cm) ^b^	102.00 (95.50–107.50)	102.00 (96.00–107.50)	0.010 *
WHR ^a^	0.82 (±0.082)	0.83 (± 0.081)	<0.001 ***
WHtR ^a^	0.502 (±0.075)	0.507 (± 0.076)	<0.001 ***

^a^ *T*-test; ^b^ Wilcoxon test; Abbreviations: BMI, body mass index; BCM, body cell mass; BFM, body fat mass; PBF, percent body fat; FFM, fat-free mass; SMM, skeletal muscle mass; TBW, total body water; ECW, extracellular water; ICW, intracellular water; VFA, visceral fat area; VFL, visceral fat level; WC, waist circumference; HC, hip circumference; WHR, waist-to-hip ratio; WHtR, waist-to-height ratio; ns = not significant *p* ≥ 0.05, * = statistically significant *p* < 0.05, ** = highly statistically significant *p* < 0.01, *** = very highly statistically significant *p* < 0.001. Values are expressed as the mean ± SD for normally distributed variables and as the median (IQR) for non-normally distributed variables.

**Table 4:** Comparison of changes in anthropometric and body composition parameters between women and men.

Variable	Δ Women (T2–T1)	Δ Men (T2–T1)	*p*-Value
Weight (kg) ^b^	0.20 (−0.80–1.10)	0.75 (−0.40–2.70)	0.019 *
BMI (kg/m^2^) ^b^	0.1 (−0.3–0.4)	0.25 (−0.2–0.8)	0.029 *
BCM (kg) ^b^	−0.1 (−0.4–0.3)	0.4 (−0.4–0.7)	0.127 ns
BFM (kg) ^b^	0.2 (−0.5–0.9)	0.8 (0.1–1.5)	0.005 **
PBF (%) ^a^	0.22 ± 1.24	0.71 ± 1.32	0.033 *
FFM (kg) ^b^	−0.1 (−0.6–0.6)	0.55 (−0.7–1.1)	0.193 ns
SMM (kg) ^b^	−0.05 (−0.4–0.3)	0.3 (−0.4–0.7)	0.127 ns
TBW (l) ^b^	−0.1 (−0.4–0.4)	0.3 (−0.6–0.9)	0.203 ns
ECW (l) ^b^	0 (−0.2–0.2)	0.1 (−0.3–0.4)	0.564 ns
ICW (l) ^b^	−0.05 (–0.3–0.2)	0.3 (−0.3–0.5)	0.146 ns
VFA (cm^2^) ^a^	1.53 ± 6.89	3.78 ± 6.35	0.064 ns
VFL (level) ^b^	0 (0–1)	0 (0–1)	0.038 *
Phase Angle (°) ^b^	0 (−0.2–0.1)	0.1 (−0.1–0.2)	0.183 ns
WC (cm) ^b^	0 (0–1)	1 (0–2)	0.020 *
HC (cm) ^b^	0 (0–1)	0 (0–1)	0.190 ns
WHR ^a^	0.005 ± 0.023	0.006 ± 0.011	0.720 ns
WHtR ^a^	0.004 ± 0.014	0.006 ± 0.008	0.260 ns

^a^ *T*-test; ^b^ Mann–Whitney test; Abbreviations: BMI, body mass index; BCM, body cell mass; BFM, body fat mass; PBF, percent body fat; FFM, fat-free mass; SMM, skeletal muscle mass; TBW, total body water; ECW, extracellular water; ICW, intracellular water; VFA, visceral fat area; VFL, visceral fat level; WC, waist circumference; HC, hip circumference; WHR, waist-to-hip ratio; WHtR, waist-to-height ratio; ns = not significant *p* ≥ 0.05, * = statistically significant *p* < 0.05, ** = highly statistically significant *p* < 0.01. Δ represents the change between post-holiday (T2) and pre-holiday (T1) measurements. Values are expressed as the mean ± SD for normally distributed variables and as the median (IQR) for non-normally distributed variables.

## Data Availability

Data are available from the corresponding author upon reasonable request.
